# Electro-acupuncture promotes the proliferation of neural stem cells and the survival of neurons by downregulating miR-449a in rat with spinal cord injury

**DOI:** 10.17179/excli2017-123

**Published:** 2017-03-23

**Authors:** Yi Zhu, Yaochi Wu, Rong Zhang

**Affiliations:** 1 Department of Acupuncture, Moxibustion, Tuina and Traumatology, Shanghai Sixth People's Hospital Affiliated to Shanghai Jiao Tong University, Shanghai, China; 2 Jinqiao Community Health Service Center, Shanghai, China

**Keywords:** spinal cord injury, electro-acupuncture, miR-449a, calcitonin gene related peptide, neuron

## Abstract

The aim of this study is to investigate the mechanism of electro-acupuncture (EA) on the recovery of injured spinal cord. Rats were randomly divided into normal control, sham-operated, SCI, SCI+EA group and T10 segment spinal cord injury (SCI) rat model was established by the modified Allen's method. After 7 days, the mRNA and protein expression of Nestin, neuron specific nuclear protein (NeuN) and calcitonin gene related peptide (CGRP) were detected by real time RT-PCR, Western blot and immunohistochemistry respectively. The protein expression of cleaved caspase 3, Bax, Bcl-2, TNF-α and IL-1β were also detected by Western blot. MicroRNA 449a (miR-449a) expression was also compared. Further, 12 SCI rats were randomly divided into EA and miR subgroups (EA + miR-449a agomir injection). The expression of Nestin, NeuN, CGRP, cleaved caspase 3, Bax, Bcl-2, TNF-α, IL-1β and miR-449a was compared. The direct interaction of miR-449a and CGRP mRNA was assessed by dual luciferase reporter assay. At day 7, compared with sham-operated group, miR-449a expression in SCI group was significantly increased (*P* < 0.05), and NeuN and CGRP mRNA and protein expression was markedly decreased (*P* < 0.05), but protein levels of Nestin, cleaved caspase 3, TNF-α, IL-1β and the ratio of Bax/Bcl-2 in SCI group were significantly increased (*P* < 0.05). The EA treatment significantly reduced miR-449a level and cleaved caspase 3, TNF-α, IL-1β level and the ratio of Bax/Bcl-2 (*P* < 0.01), but substantially increased Nestin, NeuN and CGRP expression (*P *< 0.05 or 0.01). High level of miR-449a in miR subgroup was accompanied by decreased expression of Nestin, NeuN and CGRP and increased expression of cleaved caspase 3, TNF-α, IL-1β and elevation of the ratio of Bax/Bcl-2 (*P* < 0.05), suggesting miR-449a inhibits the effects of EA on NSCs and neurons. Luciferase reporter assay showed that miR-449a bound to the 3' UTR of CGRP, and thereby regulated CGRP expression. In conclusion, EA promotes proliferation of neural stem cells and the survival of neurons by downregulation of miR-449a expression.

## Introduction

Spinal cord injury (SCI) is damage to the central nervous system (CNS) that causes temporary or permanent changes in muscle function, sensation, or autonomic function in parts of the body served by the spinal cord below the level of the lesion. To date, there is no effective treatment method for SCI primarily because most neurons in the adult CNS are terminally differentiated and cannot regenerate after injuries. A small number of neural stem cells (NSCs) have been recently identified in some regions of mature CNS such as the spinal cord. Therefore, the post-injury repair and regeneration of neurons and NSCs is crucial for the treatment of SCI. Currently, researches on SCI are mainly focusing on the regulation of genes that can stimulate the proliferation of neurons and NSCs. Nestin is a NSC-specific type IV intermediate filament protein that is required for the proper self-renewal of neural stem cells, and is closely associated with NSCs (Almazan et al., 2001[3]). Neuron-specific nuclear protein (NeuN) is a small soluble protein mainly distributed in the cytoplasm and nucleus of neurons (Mullen et al., 1992[26]). As a biomarker of NSCs, the expression level of NeuN protein indicates the ability of cell proliferation (Weyer and Schilling, 2003[36]). Calcitonin gene-related peptide (CGRP) is a 37-amino acid multifunctional neuropeptide that is widely distributed in the peripheral and central nervous system (Benarroch, 2011[6]). The protein can promote the regeneration of nerve (Blesch and Tuszynski, 2001[7]), and is involved in the post-SCI repair process (Ackery et al., 2007[1]; Zinck et al., 2007[40]). 

Acupuncture is a traditional Chinese medicine treatment developed over 2500 years ago, and has been applied in the treatment of several neurological diseases (Lee et al., 2007[22]). As an improved acupuncture method, electro-acupuncture (EA) has been successfully used in the clinical treatment of a variety of SCI-related symptoms, including loss of athletic ability, pain, muscle spasms and spinal cord hollowing (Paola and Arnold 2003[28]). EA can induce the regeneration of CGRP-positive nerve fibers in SCI rats (Yan et al., 2011[38]). EA can also increase the expression of CGRP in rat spinal cord transaction, and thereby improve the survival of damaged neurons and prevent the loss of neurons (Li et al., 2012[24]). Nevertheless, the molecular mechanism underlying the therapeutic effect of EA on SCI has not been fully clarified.

microRNAs are a class of small non-coding RNA molecules that are involved in a wide range of biological processes such as cell cycle, apoptosis, organ development, tissue regeneration, aging, and even the pathogenesis of several diseases through inhibition of target gene expression by binding to the 3'UTR region of these genes (Ambros, 2004[4]; Baranwal and Alahari, 2010[5]; Kloosterman and Plasterk, 2006[20]; Rose and Stadler, 2011[30]). As important regulators of normal nervous functions, microRNAs play vital roles in the morphological and functional changes of the nervous system (Agostini et al., 2011[2]; Wong et al., 2013[37]). Studies have found that miR-449a level is decreased in the trigeminal ganglia of migraine rats and the protein might be involved in the regulation of CGRP expression (Zhang et al., 2015[39]). In this study, the therapeutic effect and molecular mechanism of EA on SCI was investigated in a rat SCI model.

## Material and Methods

### Reagents and instruments

The trizol RNA extraction kit was purchased from Invitrogen Corp. (Carlsbad, CA, USA). Reverse transcription kit, and DreamTaq Green PCR Master Mix (2×) were purchased from Takara (Japan). Primers used in PCR amplification and miR-449a agomir (pre-miR-449a: 5'-CUGUGUGCGAUGGGUUGGCAGUGUAUUGUUAGCUGGUUGAGUAUGUAAAAGGCACCAGCUAACAUGCAACUGCUCUCCUAUUGCACAUACA-3') and the non-targeting sequence (negative control) were synthesized by GenePharma Biotech. (Shanghai, China). Protein extraction kit and BCA kit was purchased from Beyotime Institute of Biotechnology (Shanghai, China). Mouse anti-rat Nestin monoclonal antibody (Catalog No. 556309, BD Biosciences, San Jose, CA, USA), mouse anti-rat NeuN and CGRP monoclonal antibodies (ab177487 and ab139264, Abcam, Cambridge, MA, USA), cleaved caspase 3 antibody, Bax antibody, Bcl-2 antibody, TNF-α antibody and IL-1β antibody (Abcam, ab13847, ab32503, ab59348, ab6671, ab9722), mouse anti-rat GAPDH monoclonal antibody (Abcam, ab9484) and HRP-labeled rabbit anti-mouse IgGand goat anti-rabbit IgG (Abcam, ab6728 and ab6721) were used in Western blot analyses. Envision detection systems (HRP/Rabbit and mouse, K500711) used in immunohistochemistry (IHC) was purchased from Dako (Copenhagen, Denmark). Electro-acupuncture therapeutic meter (G6805-1) was manufactured by Shanghai Medical Instruments Inc. (Shanghai, China). RPMI-1640 culture medium, fetal bovine serum (FBS), penicillin-streptomycin double-resistance and trypsin were purchased from Sigma (Gbico, St. Louis, MO, USA). Quick-change site-directed mutagenesis kit was purchased from Stratagene Corporation (La Jolla, CA, USA). miR-449a mimic was synthesized by GenePharma Biotech. Firefly luciferase reporter vector pMIR-report luciferase was purchased from Ambion, Inc. (Austin, TX, USA). Lipofectamine 2000 transfection reagent was purchased from Shanghai Bioleaf Technology Co. (Shanghai, China). Opti-MEM reduced serum medium was purchased from Life Technologies, Inc. (Rockville, MD, USA). Luciferase reporter gene detector GloMax20/20 was purchased from Promega Corporation (Madison, WI, USA). Gel imager GelDoc 2000 was purchased from Bio-Rad Corporation (Hercules, CA, USA).

### Animals and grouping

Adult SD male rats weighing 180-220 g were purchased from Shanghai SLAC Laboratory Animal Co. Ltd. (Shanghai, China) and raised at room temperature. After one week, 24 rats were randomly divided into 4 groups (n = 6 each): healthy control, sham surgery, SCI surgery, and SCI+EA group in order to evaluate the therapeutic effect of EA on SCI. Further, another 12 rats were subjected to SCI surgery and randomly divided into 2 subgroups: SCI+EA subgroup and miR group (SCI+EA+miR-449aagomir) in order to investigate the regulatory role of miR-449a. SCI surgery was performed as described below in ***Establishment of SCI model*** section. After the surgery, rats in the SCI+EA group were treated with an EA device at the Jizhong and Zhiyang acupoints (Li et al., 2012[24]) 30 min/day for 7 consecutive days. The settings are as follows: coherent dilatational wave, 1 mA and a frequency of 2 Hz/100 Hz. Rats in the miR subgroup was given an intraperitoneal injection of 20 μl of 500 pmol miR-449aagomir/day for 3 days after SCI surgery in addition to the EA treatment. Sham-operated group underwent the same surgical procedure as that in SCI group except that the spinal cord was not damaged. The spinal cord of each rat was obtained at day 7 and examined by real time reverse transcription PCR (qRT-PCR) and Western blot analyses. This animal study was approved by the Research Ethic Committee at Shanghai Jiao Tong University.

### Establishment of SCI model

The T10 segment SCI rat model was constructed by the modified Allen's method (weight-drop method). Briefly, rats were fastened for 12 h before surgery. Rats lying in the prone position were given an intraperitoneal injection of 300 mg/kg chloral hydrate. The skin around T10 segment in the back was disinfected and a longitudinal incision was cut. The erector spinae muscles were separated by blunt dissection, and the lamina and spinous process was removed to expose the T10 spinal dura. A small plastic round pad was placed on the exposed spinal cord. A 20-g object was vertically dropped through a 5 cm high glass tube onto the spinal cord. The muscle and skin was sutured. The SCI model was successfully constructed as indicated by the spasmodic swing of the tail, fluttering of the body and lower limbs, and flaccid paralysis of both lower limbs. 

### Quantitative reverse transcription-polymerase chain reaction (qRT-PCR)

At day 7, rats were anesthetized by an intraperitoneal injection of 300 mg/kg chloral hydrate. The T10 segment of the spinal cord was removed and homogenized on ice. Total RNA was extracted using the RNA extraction kit. RNA concentration was determined using a spectrometer under a wavelength of 260 nm. Total RNA was reverse transcribed into cDNA using the reverse transcription kit according to the manufacturer's instructions. The Nestin, NeuN, CGRP, and GAPDH mRNA, and miR-449a was measured by qRT-PCR using cDNA as template and GAPDH and small nuclear RNA U6 (snU6) as internal control. The reaction condition: 95 °C 15 s; followed by 30 cycles of 95 °C 5 s and 60 °C 60 s. After amplification, melting curve analysis was performed: 95 °C 15 s, 60 °C 30 s, 72 °C 30 s. The sequences of primers: Nestin, forward: 5'-CGCTCAGATCCTGGAAGGTG-3', reverse: 3'-TTGGGGTCCAGAAAGCCAAG-3'; NeuN, forward: 5'-GCAGATGAAGCAGCACAGAC-3', reverse: 5'-TGAACCGGAAGGGGATGTTG-3'; CGRP, forward: 5'-CTTGCTCCTGTACCAGGCAT-3', reverse: 5'-CACACCGCTTAGATCTGGGG-3'; GAPDH, forward: 5'-CTCCTGTTCGACAGTCAGCC-3', reverse: 5'-TGGAATTTGCCATGGGTGGA-3'; miR-449a, primer for RT:5'-GTCGTATCCAGTGCAGGGTCCGAGGTATTCGCACTGGATACGACaccagc-3', forward: 5'-GGGTGGCAGTGTATTGTTA-3', reverse: 5'-CAGTGCGTGTCGTGGAGT-3'; U6, forward: 5'-GCTTCGGCAGCACATATACTAAAAT-3', reverse: 5'-CGCTTCACGAATTTGCGTGTCAT-3'. The experiment was repeated three times. Data was analyzed using the 2^-ΔCt^ method.

### Western blot analyses

The expression of Nestin, NeuN and CGRP in all groups and the protein expression of cleaved caspase 3, Bax, Bcl2, TNF-α and IL-1β in SCI+EA subgroup and miR group was compared by Western blot. Briefly, the T10 spinal cord of each rat was obtained and homogenized on ice. Total protein was extracted using protein extraction kits and quantified using a BCA kit according to the manufacture's instruction. Equal amounts of total protein (30 μg) were separated by SDS-PAGE electrophoresis and transferred to polyvinylidene difluoride membranes. The membrane was blocked in TBS buffer containing 5 % skim milk and 0.1 % Tween20 at room temperature for 2 h, and incubated with the appropriate primary antibody (1:1000) overnight at 4 °C. The membrane was washed with TBST and incubated with HRP-labeled rabbit anti-rat secondary antibodies (1:5000) at 37 °C for 2 h. The membranes were washed thoroughly with TBST for 5 min each and subjected to ECL detection. The intensity of bands was detected by a gel imager. The gray value of bands was analyzed by Image Lab 2.0 software (Bio-Rad Laboratories).

### Immunohistochemical staining

The protein quantity and distribution of Nestin, NeuN and CGRP was detected using immunohistochemical staining with EnVision detection systems. Briefly, paraffin sections were dewaxed and hydrated with tradition methods, then were incubated with 0.3 % H_2_O_2_ for 10 min to block the activity of endogenous peroxidase followed by hot antigen repair. These sections were incubated in serum from nonimmune animals (rabbit or mouse) for 20 min at 37 °C before incubated with primary antibodies at 4 °C for overnight. Then sections were incubated with EnVision reagent (HRP-R/M) at 37 °C for 30 min and visualized with DAB reagent. Finally, sections were counterstained with hematoxylin and counted the positive cells by microscope.

### Cell culture

Human embryonic kidney cells (HEK) 293T cells were purchased from the Cell Bank of Type Culture Collection of Chinese Academy of Sciences (CCCAS, Shanghai, China). Cells were cultured in RPMI-1640 medium containing 10 % FBS, 50 U/mL penicillin and 50 μg/mL streptomycin at 37 °C in an incubator with 5 % CO_2_. Cells in the logarithmic growth phase were used for subsequent luciferase reporter assay.

### Luciferase reporter assay

The CGRP gene (alias: CALCA) was analyzed using the online tool (http://www.targetscan.org) to predict the 3'UTR sequence for miRNA-449a. The primers were designed using Primer Premier 5.0 software and synthesized by GenePharma Biotech. The sense and antisense primer was 5'-CGCGGATCC ACTCTGCTAAACCTCAAGGGG-3', and 5'-CCGGAATTC GTCACAACATTACCATGTGTCCC-3', which separately contained a BamHI restriction site and an EcoRI restriction site. Total RNA of HEK 293T cells was extracted using the trizol kit and reverse transcribed into cDNA using the reverse transcription kit according to the manufacturer's instructions. The target sequence was amplified using cDNA as template. The amplified fragment was isolated by gel electrophoresis, extracted, ligated to T vector, and transformed into *Escherichia coli*. Clones were selected and sequenced to obtain the CGRP-UTR fragment. Mutagenesis was performed at the miR-449a binding site using the Quick-change site-directed mutagenesis kit. The sequence of the mutated fragment CGRP-UTR-mut was confirmed by sequencing. The luciferase reporter plasmids pMIR-CGRP-UTR and pMIR-CGRP-UTR-mut were constructed using the firefly luciferase reporter vector pMIR-report luciferase. HEK 293T cells at 70 % confluence were respectively transfected with 500 ng of pMIR-CGRP-UTR, pMIR-CGRP-UTR-mut, and blank control pMIR, and 10 nM of miR-449a mimics or negative control using Lipofectamine 2000 transfection kit and cultured in Opti-MEM reduced serum medium and the luciferase activity was measured using dual luciferase assay kit following the manufacturer's instructions. The relative luciferase activity (RLA) was calculated as the ratio of firefly luciferase activity (Ff) to renilla luciferase activity (Rn).

### Statistical analyses

All experiments were repeated three times, and data were expressed as mean ± standard deviation. Statistical analyses were performed using SPSS 11.0 (SPSS Inc, Chicago, IL, USA). Differences among groups were analyzed by one-way ANOVA followed by Tukey's test. *P* values smaller than 0.05 were considered statistically significant.

## Results

### Effects of EA on Nestin, NeuN, CGRP and miR-449a expression

**Figure 1:**
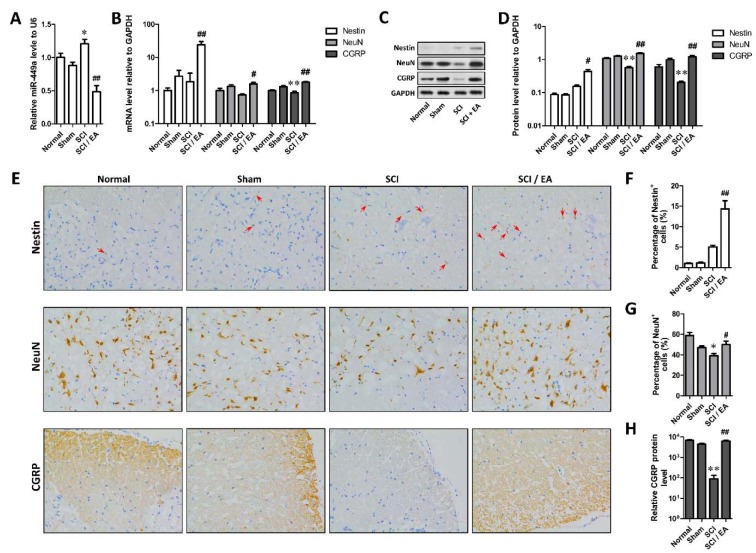
Electro-acupuncture downregulated miR-449a and promote the expression of NSC biomarker Nestin and neuron biomarker NeuN. A. Differences of miR-449a among groups; B. MRNA levels of Nestin, NeuN and CGRP in each group; C. Protein levels of Nestin, NeuN and CGRP detected by Western blotting in each group; D. Densitometric analysis results of Western blots; E. Representative immunohistochemical stain of Nestin, NeuN and CGRP in ventral horn of spinal cord; F-H. Quantification of immunohistochemical stain of Nestin (F), NeuN (G) and CGRP (H). *P < 0.05, **P<0.01, compared with Sham group; ^#^P < 0.05, ^##^P < 0.01, compared with SCI group.

The miR-449a expression in SCI group was significantly increased compared with that in sham-operated group (*P* < 0.05), whereas miR-449a expression was significantly reduced after EA treatment (*P *< 0.01, Figure 1A(Fig. 1)). As shown in Figure 1B-D(Fig. 1), the expression of NeuN and CGRP protein in SCI group was significantly decreased compared with that in sham-operated group (*P* < 0.05 or 0.01). While the expression of Nestin protein in SCI group was to some extent higher than that in sham-operated group. We found no significant difference in Nestin expression between the two groups. When compared with SCI group, the Nestin, NeuN and CGRP mRNA and protein expression in SCI+EA group was significantly increased (*P* < 0.05 or 0.01). The immunohistochemical results also showed that Nestin protein level was raised to some degree, but NeuN and CGRP protein levels were markedly declined in SCI group (*P* < 0.01 or *P* < 0.05), which were all raised in SCI+EA group (*P* < 0.05 or 0.01) (Figure 1E-H(Fig. 1)), suggesting that SCI might elevate the Nestin level but reduce the NeuN and CGRP level in spinal cord of rats, and EA treatment after SCI would elevate the level of Nestin, NeuN and CGRP in ventral horn of spinal cord of rats.

### EA inhibit the SCI-induced increase of cleaved caspase 3, Bax/Bcl-2 ratio, TNF-α and IL-1β in spinal cord of rats

**Figure 2:**
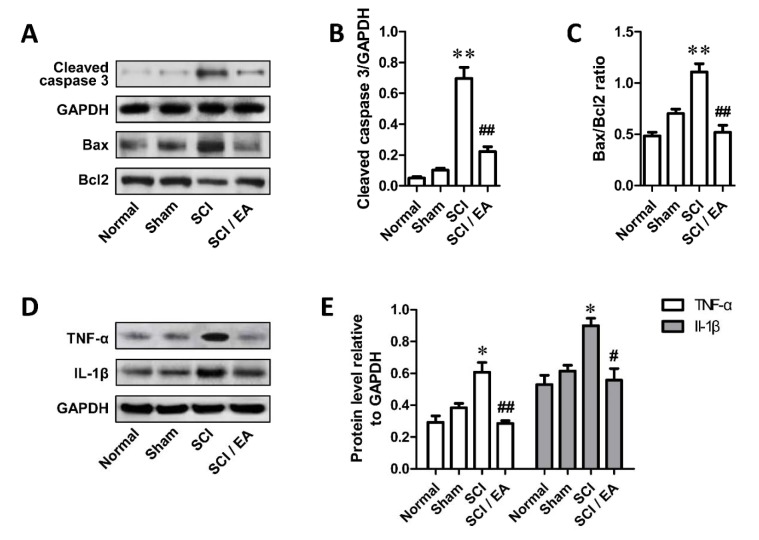
Electro-acupuncture inhibited cell apoptosis and inflammation in ventral horn of injured spinal cord. A. Protein level of cleaved caspase 3, Bax and Bcl2 measured with Western blotting; B, C. Relative level of cleaved caspase 3 and ratio of Bax to Bcl2 quantified by densitometric analysis; D. Relative level of inflammatory factors TNF-α and IL-1β measured with Western blotting; E. Densitometric analysis results of the protein levels of TNF-α and IL-1β. *P < 0.05, **P < 0.01, compared with Sham group; ^#^P < 0.05, ^##^P < 0.01, compared with SCI group.

The protein levels of cleaved caspase 3, Bax, Bcl-2, TNF-α and IL-1β in ventral horn of spinal cord of rats were detected using Western blot and the results were shown in Figure 2(Fig. 2). Densitometric analysis showed that relative protein level of cleaved caspase 3, TNF-α and IL-1β and the ratio of Bax to Bcl-2 were all significantly elevated in SCI group than that in sham-operated group (*P* < 0.05 or 0.01). But EA treatment after SCI could attenuate the promotion (*P* < 0.05 or 0.01) (Figure 2B, 2C, 2E(Fig. 2)). These results indicate that EA could suppress the SCI-stimulated elevation of cleaved caspase 3, TNF-α and IL-1β, and Bax/Bcl-2 ratio; EA might inhibit cell apoptosis and inflammation in ventral horn of injured spinal cord of rats.

### Effect of miR-449a agomir on EA-stimulated Nestin, NeuN and CGRP expression and EA-reduced level of cleaved caspase 3, TNF-α and IL-1β and the ratio of Bax to Bcl-2

**Figure 3:**
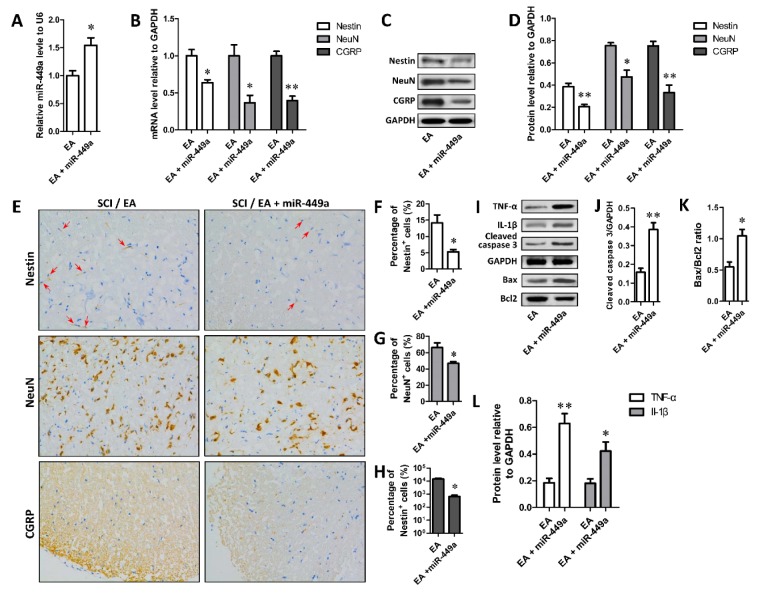
MiR-449 mediated the promotive effect of EA on the expression of NSC biomarker Nestin and neuron biomarker NeuN. A. miR-449a agomir treatment (20 μl 500 pmol, intraperitoneal injection, for 3 days after SCI) promoted miR-449a level; B-D. miR-449a agomir treatment significantly restored the elevated mRNA (B) and protein (C, D) levels of Nestin, NeuN and CGRP by EA; E. Representative immunohistochemical stain of Nestin, NeuN and CGRP in ventral horn of spinal cord; F-H. Quantification of immunohistochemical stain of Nestin (F), NeuN (G) and CGRP (H); I-L. Protein level of TNF-α, IL-1β, cleaved caspase 3, Bax and Bcl2 measured with Western blotting, and quantified based on densitometry analysis. *P < 0.05, **P < 0.01, compared with SCI+EA group

Further, the regulation of miR-449a on EA-stimulated Nestin, NeuN and CGRP expression was assessed by comparing rats receiving EA treatment and EA+miR-449a agomir treatment. As shown in Figure 3A(Fig. 3), miR-449a expression in miR group was significantly higher than that in EA group (*P* < 0.05). Moreover, the results of real time PCR, Western blot and immunohistochemical staining were also shown that the expression of Nestin, NeuN and CGRP mRNA and protein in miR group was significantly reduced compared with EA group (*P* < 0.05 or 0.01) (Figure 3B-H(Fig. 3)). In addition, protein expression detection of cleaved caspase 3, Bax, Bcl-2, TNF-α and IL-1β showed that the relative expression of cleaved caspase 3, TNF-α and IL-1β proteins and Bax/Bcl-2 ratio in miR group were obviously higher than that in EA group (*P* < 0.05 or 0.01) (Figure 3I-L(Fig. 3)). These results suggest that high level of miR-449a could suppress the effect of EA on the expression of Nestin, NeuN, CGRP, cleaved caspase 3, Bax, Bcl-2, TNF-α and IL-1β in spinal cord of rats.

### miR-449a regulates CGRP expression by directly binding to its 3'UTR

**Figure 4:**
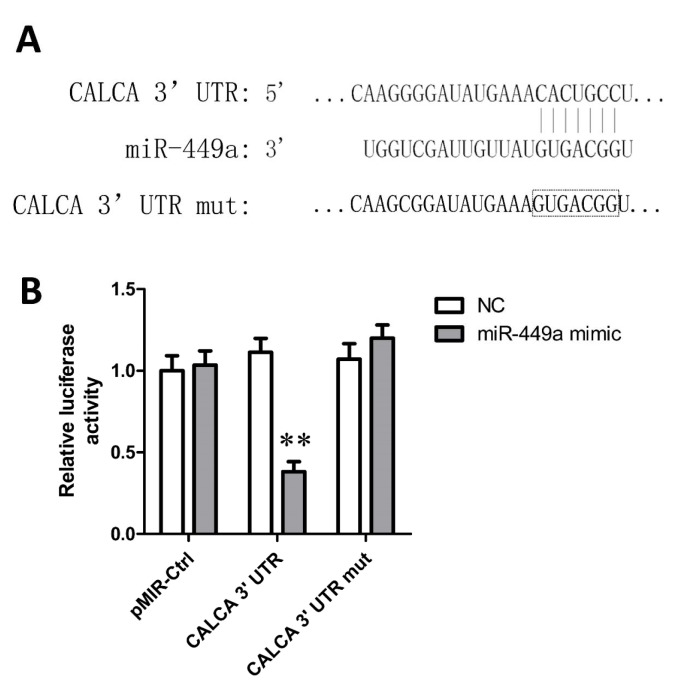
Luciferase reports assay showed a direct target site of miR-449a in 3' UTR of CGRP mRNA. A. Predicted binding site between miR-449a and CGRP 3' UTR and the contrived mutant site in CGRP 3' UTR; B. miR-449a mimic targeted at CGRP 3' UTR and degraded luciferase mRNA. *P < 0.05, **P < 0.01, compared with negative control (NC).

CGRP was predicted to have a binding site in the 3'UTR sequence for miRNA-449a (Figure 4A(Fig. 4)). The regulation of miR-449a on the CGRP expression was further determined by Luciferase reporter assay. When compared with negative control group, miR-449a mimics significantly reduced the RLA in HEK 293T cells transfected with pMIR- CGRP-UTR (RLA = 0.47, *P* < 0.01), but did not affect the RLA in pMIR-CGRP-UTR-mut group (Figure 4B(Fig. 4)), indicating that miR-449a could recognize and bind to the 3'-UTR of CGRP mRNA and regulate the CGRP expression. 

## Discussion

Spinal cord injury is an extremely serious nerve damage that can lead to severe loss of body function. However, the efficacy of current treatment methods is largely limited (Calancie et al., 2005[10]; Thuret et al., 2006[34]). Studies have suggested that the post-SCI functional recovery of the nervous system is affected by both primary injury and local nerve cell loss due to secondary cell atrophy, necrosis and apoptosis, and the repair and regeneration of nerve cells, especially neurons and NSCs, is crucial for the recovery of neurological function (Hagg and Oudega, 2006[18]; Kwon and Tetzlaff, 2001[21]).

It has been known that NSCs play an important role in the repair of SCI (Frisen et al., 1995[15]; Gould and Tanapat, 1997[17]). Nestin, the NSC biomarker is highly expressed in actively proliferating NSCs. The expression of Nestin is reduced in mature neurons and glial cells, but is increased in injured nervous tissues (Lendahl et al., 1990[23]; Ruehm et al., 2001[31]). Therefore, the changes in NSCs in SCI can be reflected by the expression of Nestin. As a specific marker for mature neurons, NeuN is distributed in most of the neurons in the central nervous system, and has been widely used to identify bone marrow-derived neurons, neuronal nuclei sorting, etc. (Sharp et al., 2002[32]). Studies have found that CGRP expression is upregulated in peripheral nerve injuries (Jungnickel et al., 2005[19]). Moreover, constant expression of CGRP is closely associated with the regeneration of the nerve (Snyder et al., 2002[33]). The mechanism might be related to its promotive effect on the blood supply to the nervous system (Chang et al., 2014[12]). In this study, the therapeutic effect of EA on injured spinal cord was evaluated in SCI rats. It was found that EA stimulated the expression of Nestin, NeuN and CGRP, suggesting that EA promoted the regeneration of neurons and NSCs, which was consistent with previous studies (Li et al., 2012[24]; Yan et al., 2011[38]).

Caspase-3, an inactive form, is located in the cytoplasm and activated by proteolysis to produce the cleaved caspase-3 (Porter and Janicke, 1999[29]). Cleaved caspase-3 is an executioner caspase and promotes apoptosis, so the level of cleaved caspase-3 indicates the level of cellular apoptosis (Chakravarti et al., 2004[11]). Bax, a proapoptotic protein, can bind to the antiapoptotic protein Bcl-2 to regulate the level of cellular apoptosis, increased expression of Bcl-2 inhibits apoptosis and increased expression of Bax promotes apoptosis (Mo et al., 2016[25]). TNF-α and IL-1β are pro-inflammatory molecules, can cause and promote inflammation (Galli et al., 2008[16]). TNF-α and IL-1β are also involved in apoptosis and NSCs have been proved to inhibit the expression of TNF-α and IL-1β in SCI (Cheng et al., 2016[14]). Our study showed EA could suppress the SCI-induced elevation of cleaved caspase 3, Bax/Bcl-2 ratio, TNF-α and IL-1β, which indicates that EA may inhibit apoptosis and inflammation through attenuating the mature of caspase-3 and expression of TNF-α and IL-1β and the ratio of Bax/Bcl-2.

MiR-449a is a recently identified member of the conserved miR-449 family. Studies have shown that miR-449 a/b level in liver cancer, bladder cancer, prostate cancer and gastric cancer is markedly lower compared with normal tissues, and exogenous miR-449 a/b expression can inhibit cell proliferation, and induce cell cycle arrest, senescence and apoptosis (Bou Kheir et al., 2011[8]; Buurman et al., 2012[9]; Chen et al., 2012[13]; Noonan et al., 2009[27]). miR-449 may silence the target gene by binding to its 3'-UTR region (Tsujiura et al., 2010[35]). Nevertheless, although miR-449a is well studied in a variety of cancers, its regulatory role in post-SCI neurological functional recovery has seldomly been studied. miRNA agomir is a synthetic RNA duplex with certain chemical modifications that is developed for stimulating *in vivo* miRNA activity. In the current study, SCI rats were treated by EA combined with intraperitoneal injections of miR-449a agomir. It was found that miR-449a agomir upregulated the miR-449a level in these rats, which inhibited the high expression of Nestin, NeuN and CGRP. The result suggested that miR-449a mediated the stimulative effect of EA on Nestin, NeuN and CGRP expression. High level of miR-449a promoted the mature of caspase-3 and expression of TNF-α and IL-1β and the ratio of Bax/Bcl-2 which were suppressed by EA in ventral horn of injured spinal cord of rats. Furthermore, the regulatory mechanism of miR-449a on CGRP gene expression was also investigated by luciferase reporter assay. It was found that miR-449a mimic specifically bound to the 3'UTR of CGRP mRNA, revealing that miR-449a directly regulated the CGRP gene expression on the post-transcription level. 

In summary, EA promotes the expression of neuronal markers Nestin, NeuN and CGRP and inhibits cellular apoptosis and inflammation in SCI rats. CGRP mRNA is a direct target for miR-449a and the stimulation of EA on CGRP level is achieved by downregulation of miR-449a expression. The current study has shed lights on the molecular mechanism of EA in the treatment of SCI and will provide new ideas for the development of more effective treatment strategies. For instance, miR-449a antagonist may be used in combination with EA to improve the treatment outcome of SCI. Nevertheless, such speculation needs to be validated in future studies.

## Acknowledgements

The Construction Project of a 3-year action plan (2014-2016) in development of Traditional Chinese Medicine in Shanghai (Grant No: ZY3-JSFC-1-1008).

## Conflict of interest

The authors declare that they have no conflict of interest.

## References

[1] Ackery AD, Norenberg MD, Krassioukov A (2007). Calcitonin gene-related peptide immunoreactivity in chronic human spinal cord injury. Spinal Cord.

[2] Agostini M, Tucci P, Steinert JR, Shalom-Feuerstein R, Rouleau M, Aberdam D (2011). MicroRNA-34a regulates neurite outgrowth, spinal morphology, and function. Proc Natl Acad Sci U S A.

[3] Almazan G, Vela JM, Molina-Holgado E, Guaza C (2001). Re-evaluation of nestin as a marker of oligodendrocyte lineage cells. Microsc Res Tech.

[4] Ambros V (2004). The functions of animal microRNAs. Nature.

[5] Baranwal S, Alahari SK (2010). miRNA control of tumor cell invasion and metastasis. Int J Cancer.

[6] Benarroch EE (2011). CGRP: sensory neuropeptide with multiple neurologic implications. Neurology.

[7] Blesch A, Tuszynski MH (2001). GDNF gene delivery to injured adult CNS motor neurons promotes axonal growth, expression of the trophic neuropeptide CGRP, and cellular protection. J Comp Neurol.

[8] Bou Kheir T, Futoma-Kazmierczak E, Jacobsen A, Krogh A, Bardram L, Hother C (2011). miR-449 inhibits cell proliferation and is down-regulated in gastric cancer. Mol Cancer.

[9] Buurman R, Gurlevik E, Schaffer V, Eilers M, Sandbothe M, Kreipe H (2012). Histone deacetylases activate hepatocyte growth factor signaling by repressing microRNA-449 in hepatocellular carcinoma cells. Gastroenterology.

[10] Calancie B, Molano MR, Broton JG (2005). Epidemiology and demography of acute spinal cord injury in a large urban setting. J Spinal Cord Med.

[11] Chakravarti A, Zhai G, Suzuki Y, Sarkesh S, Black PM, Muzikansky A (2004). The prognostic significance of phosphatidylinositol 3-kinase pathway activation in human gliomas. J Clin Oncol.

[12] Chang HM, Wei IH, Tseng CY, Lue JH, Wen CY, Shieh JY (2004). Differential expression of calcitonin gene-related peptide (CGRP) and choline acetyltransferase (ChAT) in the axotomized motoneurons of normoxic and hypoxic rats. J Chem Neuroanat.

[13] Chen H, Lin YW, Mao YQ, Wu J, Liu YF, Zheng XY (2012). MicroRNA-449a acts as a tumor suppressor in human bladder cancer through the regulation of pocket proteins. Cancer Lett.

[14] Cheng Z, Zhu W, Cao K, Wu F, Li J, Wang G (2016). Anti-inflammatory mechanism of neural stem cell transplantation in spinal cord injury. Int J Mol Sci.

[15] Frisen J, Johansson CB, Torok C, Risling M, Lendahl U (1995). Rapid, widespread, and longlasting induction of nestin contributes to the generation of glial scar tissue after CNS injury. J Cell Biol.

[16] Galli SJ, Tsai M, Piliponsky AM (2008). The development of allergic inflammation. Nature.

[17] Gould E, Tanapat P (1997). Lesion-induced proliferation of neuronal progenitors in the dentate gyrus of the adult rat. Neuroscience.

[18] Hagg T, Oudega M (2006). Degenerative and spontaneous regenerative processes after spinal cord injury. J Neurotrauma.

[19] Jungnickel J, Klutzny A, Guhr S, Meyer K, Grothe C (2005). Regulation of neuronal death and calcitonin gene-related peptide by fibroblast growth factor-2 and FGFR3 after peripheral nerve injury: evidence from mouse mutants. Neuroscience.

[20] Kloosterman WP, Plasterk RH (2006). The diverse functions of microRNAs in animal development and disease. Dev Cell.

[21] Kwon BK, Tetzlaff W (2001). Spinal cord regeneration: from gene to transplants. Spine.

[22] Lee H, Park HJ, Park J, Kim MJ, Hong M, Yang J (2007). Acupuncture application for neurological disorders. Neurol Res.

[23] Lendahl U, Zimmerman LB, McKay RD (1990). CNS stem cells express a new class of intermediate filament protein. Cell.

[24] Li WJ, Li SM, Ding Y, He B, Keegan J, Dong H (2012). Electro-acupuncture upregulates CGRP expression after rat spinal cord transection. Neurochem Int.

[25] Mo ZT, Li WN, Zhai YR, Gong QH (2016). Icariin attenuates OGD/R-induced autophagy via Bcl-2-dependent cross talk between apoptosis and autophagy in PC12 cells. Evid Based Complement Alternat Med.

[26] Mullen RJ, Buck CR, Smith AM (1992). NeuN, a neuronal specific nuclear protein in vertebrates. Development.

[27] Noonan EJ, Place RF, Pookot D, Basak S, Whitson JM, Hirata H (2009). miR-449a targets HDAC-1 and induces growth arrest in prostate cancer. Oncogene.

[28] Paola FA, Arnold M (2003). Acupuncture and spinal cord medicine. J Spinal Cord Med.

[29] Porter AG, Janicke RU (1999). Emerging roles of caspase-3 in apoptosis. Cell Death Differ.

[30] Rose D, Stadler PF (2011). Molecular evolution of the non-coding eosinophil granule ontogeny transcript. Front Genet.

[31] Ruehm SG, Zimny K, Debatin JF (2001). Direct contrast-enhanced 3D MR venography. Eur Radiol.

[32] Sharp FR, Liu J, Bernabeu R (2002). Neurogenesis following brain ischemia. Brain Res Dev Brain Res.

[33] Snyder SK, Byrnes KR, Borke RC, Sanchez A, Anders JJ (2002). Quantitation of calcitonin gene-related peptide mRNA and neuronal cell death in facial motor nuclei following axotomy and 633 nm low power laser treatment. Lasers Surg Med.

[34] Thuret S, Moon LD, Gage FH (2006). Therapeutic interventions after spinal cord injury. Nat Rev Neurosci.

[35] Tsujiura M, Ichikawa D, Komatsu S, Shiozaki A, Takeshita H, Kosuga T (2010). Circulating microRNAs in plasma of patients with gastric cancers. Br J Cancer.

[36] Weyer A, Schilling K (2003). Developmental and cell type-specific expression of the neuronal marker NeuN in the murine cerebellum. J Neurosci Res.

[37] Wong HK, Veremeyko T, Patel N, Lemere CA, Walsh DM, Esau C (2013). De-repression of FOXO3a death axis by microRNA-132 and -212 causes neuronal apoptosis in Alzheimer's disease. Hum Mol Genet.

[38] Yan Q, Ruan JW, Ding Y, Li WJ, Li Y, Zeng YS (2011). Electro-acupuncture promotes differentiation of mesenchymal stem cells, regeneration of nerve fibers and partial functional recovery after spinal cord injury. Exp Toxicol Pathol.

[39] Zhang CL, Liu D, Yan YH, Guan XY, Wan Q (2015). The microRNAs that may regulate calcitonin gene-related peptide expression in the trigeminal ganglion in a rat model of migraine. Chin J Pain Med.

[40] Zinck ND, Rafuse VF, Downie JW (2007). Sprouting of CGRP primary afferents in lumbosacral spinal cord precedes emergence of bladder activity after spinal injury. Exp Neurol.

